# Socio-demographic and disease related characteristics associated with unplanned emergency department visits by cancer patients: a retrospective cohort study

**DOI:** 10.1186/s12913-019-4509-z

**Published:** 2019-09-06

**Authors:** Polly H. Dufton, Allison Drosdowsky, Marie F. Gerdtz, Mei Krishnasamy

**Affiliations:** 1 Department of Nursing & Centre for Cancer Research, The University of Melbourne, Parkville, VIC Australia; 2 The Olivia Newton John Cancer, Wellness and Research Centre, Heidelberg, VIC Australia; 3 Department of Cancer Experiences Research, Sir Peter McCallum Cancer Centre, Parkville, VIC Australia; 4 Victorian Comprehensive Cancer Centre, Parkville, VIC Australia

**Keywords:** Cancer, Oncology, Antineoplastics, Chemotherapy, Emergency department, Risk reduction, Andersen’s behavioral model of health service utilization

## Abstract

**Background:**

Emergency department (ED) presentations made by patients having cancer treatment are associated with worth outcomes. This study aimed to explore the socio-demographic and disease related characteristics associated with ED presentation, frequent ED presentations, and place of discharge for cancer patients receiving systemic cancer therapies in the ambulatory setting.

**Methods:**

This was a single site, retrospective observational cohort design. Hospital data for patients treated in the Day Oncology Unit of a large public tertiary hospital in Melbourne, Australia between December 2014 and November 2017 were extracted from clinical databases and retrospectively matched to ED attendance records. Andersen’s Behavioral Model of Health Service Utilisation provided the conceptual framework for exploring associations between socio-demographic and disease characteristics and ED use.

**Results:**

A total of 2638 individuals were treated in the Day Oncology Unit over the study dates. Of these, 1182 (45%) made an unplanned ED presentation within 28 days of receiving systemic cancer therapy. One hundred and twenty-two (12%) patients attended the ED on two or more occasions within 28 days; while 112 (10%) patients attended the ED four or more times (within 28 days of receiving systemic cancer therapy) within any given 12 month period. Being born outside of Australia was independently related to making an unplanned ED presentation within 28 days of receiving anti-cancer therapy (*p* < .01) as was being diagnosed with head and neck (*p* = .03), upper gastrointestinal (*p* < .001), colorectal (*p* < .001), lung (*p* < .001), skin (*p* < .001) or breast cancer (*p* = .01).

**Conclusions:**

This study identified a subgroup of cancer patients for whom an ED presentation is more likely. Better understanding of socio-demographic and disease related characteristics associated with the risk of an ED presentation may help inform targeted follow up of patients, to mitigate potentially avoidable ED presentation and optimize outcomes of care.

## Introduction

Patients who present to an Emergency Department (ED) during their cancer treatment are known to have poorer outcomes than those who do not, often experiencing lengthy admission post ED presentation, treatment dose reductions and treatment delays [1–3]. The ED often provides cancer patients access to unscheduled medical care or access to specialist oncology advice for symptoms they are experiencing [4]. At a system level, unplanned ED presentations place substantial burden on hospital resources [5, 6]. The high rates of unplanned ED presentations made by cancer patients are of increasing concern to the international health care community and efforts have been made to develop strategies to reduce ED presentations that may be potentially avoidable [7].

There is a substantial amount of variance reported for factors associated with unplanned ED presentations by cancer patients. This may be as a result of the heterogeneity within studies, which have included patients on a range of treatments, patients receiving care in acute and ambulatory settings, which have assessed different time frames for readmission and have highly variable study settings representing different models of care and available support services [1, 5, 8–10]. What is known however, is that patients receiving systemic cancer treatment (that is chemotherapy, targeted therapy and immunotherapy) in ambulatory settings have high rates of hospital representation [11–13] many of which may be mitigated through targeted screening or assessment [14, 15]. Recently, a systematic review of cancer patients’ use of EDs by Lash et al.*,* identified a number of social determinants likely to be associated with ED presentation for people with cancer. These included ethnicity, fiscal resources, social policies of the local community, marital status, health literacy, education and income [16]. Social determinants of health have been shown not only to increase the likelihood of cancer, but also to dictate a person’s ability to access, navigate and benefit from use of the health care system [17].

A number of interventions aimed at addressing potentially avoidable unplanned, cancer-related ED presentations have been published. In particular, there has been a focus on timely identification and response to patient-reported problems through patient navigation and care coordination models. In the U.K., U.S. and Canada, specialist oncology nurse-led models of care have been implemented to provide patients with timely access to telephone consultation, advice and support following receipt of systemic cancer therapies [18, 19]. These services focus on strengthening patient education for self-management, timely assessment and response to cancer symptoms or treatment side-effects, and helping patients navigate the health care system. They have been shown to reduce ED presentations and subsequent inpatient length of stay [20–23]. However, few studies have described methods to pro-actively identify cancer patients who are most at risk of making unscheduled ED presentations after receiving cancer treatment, and who may subsequently be targeted for follow up to reduce their risk of an unplanned ED presentation and consequent poorer health outcomes [20].

### Aims

This study aimed to explore socio-demographic (patient) and disease related characteristics of cancer patients presenting to an ED within 28 days of receiving infusional, systemic anti-cancer therapy in a Day Oncology Unit (DOU), in order to identify those at greater risk of making an unplanned ED presentation, and to make recommendations for risk stratified models of follow up care.

For the purpose of our study, infusional, systemic anti-cancer therapy (SACT) was defined as: any type of treatment that uses drugs or other substances to travel through the bloodstream to identify and attack cancer cells, either by blocking or damaging certain enzymes, proteins, or other molecules involved in the growth and spread of cancer cells; or modulation of the immune system; or by stopping normal and cancer cells dividing or by directly causing cell death [24].

The objectives of the study were to explore socio-demographic and disease related characteristics associated with:

1. Making one or more ED presentation within 28 days of receiving SACT;
2. Making multiple ED presentations within 28 days of receiving SACT;
3. Making frequent (≥ 4 within any 12 month period) ED presentations within 28 days of receiving SACT;
4. An ED discharge disposition (i.e. admission or discharge) of primary place of residence.

Andersen’s Behavioural Model of Health Service Utilisation was used to guide the exploration of factors that may be associated with ED utilisation in this study. The model has been widely used to explain variations in health service utilisation in community, acute and emergency care settings [25].

## Methods

### Setting

This study was conducted at a large, public tertiary hospital in Victoria, Australia which includes a dedicated cancer centre, with two oncology/hematology inpatient wards as well as a dedicated palliative care unit. The centre also consists of a radiotherapy unit and a DOU that has 18 treatment chairs and treats approximately 70 patients per month. The cancer centre services a complex case mix of patients and includes a large cancer clinical trials unit and provision for allogeneic hematopoietic stem cell transplantation. The hospital has a large Level II ED and trauma centre. The ED contains a Short Stay Unit (SSU) where patients may be admitted for further care following an ED presentation for a period of no longer than 24 h [26].

During business hours at the cancer centre patients who experience or have concerns about symptoms or side-effects of treatment are directed to contact a Clinical Nurse Consultant (hematological cancers, or patients receiving oral therapies or concurrent chemotherapy/radiotherapy in solid tumours) or call the DOU to speak to one of the senior oncology nurses. After hours, patients are directed to call the oncology inpatient wards for symptom support or advice. The DOU does not have a dedicated space for ad-hoc patient reviews, and commonly patients are provided with telephone advice, advised to attend their General Practitioner, or present to the ED.

### Sample

Patients were eligible for inclusion if they received SACT in the DOU between 1 December 2014 and 30 November 2017. Patients treated with subcutaneous treatments or who received supportive therapies only (for example, zoledronic acid) were not eligible and therefore not included in the final dataset.

All episodes of care (that is, any attendance for SACT) in the DOU during the study period were linked to any ED presentation at the study site that fell within 28 days from the date of discharge from the DOU. Episodes of care that were not allocated an ICD-10 code were excluded. The final dataset included specific variables available from the Victorian Admitted Episodes Dataset (VAED) and the Victorian Emergency Minimum Dataset (VEMD). The VAED and VEMD are comprehensive datasets that collect details about the causes, effects and nature of illnesses as well as details on health service use for all public hospitals in the State of Victoria [27, 28].

Demographic and clinical variables collected included age, gender, postcode, country of birth, marital status, preferred language, interpreter required, Aboriginal or Torres Strait Islander status, usual accommodation, attendance source, time of triage, ED length of stay, mode of arrival to ED, triage category using the Australasian Triage Scale, ED discharge destination and inpatient length of stay. Cancer type was classified according to the International Classification of Diseases and Related Health Problems, 10th Revision, Australian Modification (ICD-10-AM) [29]. Data were screened for outliers such as those who made a high number of ED presentations, and who had an exceptionally long ED or inpatient length of stay recorded and were verified through a manual chart review.

### Data analysis

Data were analyzed using IBM SPSS version 25.0 (Chicago, USA). The data analysis framework was developed to address the four research questions set for the study. Univariate analysis was performed, and continuous data reported as mean and standard deviation (SD) unless otherwise stated. Categorical data are reported as frequency and percentage. Country of birth, primary language and use of interpreter for in-hospital communication were highly correlated and thus, only country of birth was included in regression modelling.

Marital status was collapsed from six categories to two categories “married, de facto” and “single, divorced, widowed” representing those reporting having a significant other, or not. Cancer diagnoses were classified according to the ICD-10 coding and were collapsed from 14 categories into 10 with thyroid, bone and soft tissue, rare, gynecological, skin – non-melanoma and unknown primary grouped into ‘other’ cancers. Hematological cancers were used as the reference category, as they effect both males and females equally, encompass a variety of individual diagnoses and associated symptoms, are the largest tumor stream treated at the cancer center, and had the highest number of unplanned ED presentations.

The Socio-Economic Indexes for Areas (SEIFA) is a product developed by the Australian Bureau of Statistics which ranks areas in Australia according to relative socio-economic advantage and disadvantage [30]. SEIFA codes for all included patient records were ranked from lowest to highest and organized into quintiles. The top 20% represents those with the highest socioeconomic status, those that fall between 40 and 80% represent the middle bracket, and the bottom 40% represent those with the lowest socioeconomic status.

Destination from ED was grouped into two categories (discharged to place of primary residence or admitted to the inpatient area). Country of birth was collapsed in to two categories, born in Australia or born outside of Australia.

A binary outcome was defined for all multivariate logistic regression models as ED presentation/s “Yes/No”.

Adjusted odds ratios (AORs), 95% confidence intervals (95% CI) and *p* values are provided for variables associated with ED presentation and place of discharge. *p* values at < .05 were considered statistically significant. Effect size was calculated on AORs [31].

## Results

A total of 2638 individuals were treated for cancer in the DOU during the study period, of which 1182 (45%) made one or more unplanned ED presentation within 28 days of receiving SACT. Overall, there were 2310 unplanned ED presentations made within 28 days of receiving treatment.

One hundred and forty-four patients (12%) presented to the ED on two or more occasions within any given 28 day period after receiving SACT. The average number of ED presentations made within any given 28 day period after receiving treatment was 1.1 and ranged from one (*n* = 1038, 88%) to five (n = 1, 0.1%). One hundred and twelve (10%) patients were classified as frequent attenders because they had presented to the ED four or more times within any given 12 month period during the study. Most ED presentations (*n* = 1341, 58%) resulted in the patient being admitted to the inpatient area for further care, with a further 12% (*n* = 271) being admitted to the SSU. The remaining 30% of ED episodes (*n* = 698) resulted in the patient being discharged to their place of primary residence.

### Patient sample characteristics

Patient demographics are shown in Table 1. Where individual demographics were used in analyses, age was taken at the first DOU episode of care. Where episodes were used for analyses, age was recalculated at each DOU episode of care accordingly.

**Table 1:** Comparison of demographic factors for patients who did and did not make an ED presentation (*N* = 2638)

	ED presentation absent (*n* = 1456)		ED presentation present (*n* = 1182)		Total (*N* = 2638)	
Age (mean SD)	60.83 (13.72)		62.46 (14.14)		61.56 (13.93)	
Gender (n %)						
Male	797	54.74	626	52.96	1423	53.94
Female	659	45.26	556	47.04	1215	46.06
ATSI (n %)						
No	1286	88.32	1116	94.42	2402	91.05
Yes	5	0.34	4	0.34	9	0.34
Country of Birth (n %)						
Australia	868	59.62	631	53.38	1499	56.82
Country other than Australia	527	36.20	548	46.36	1075	40.75
Preferred Language (n %)						
English	1249	85.78	989	83.67	2238	84.84
Language other than English	126	8.65	174	14.72	300	11.37
SEIFA (VIC) (n %)						
0–40%	424	29.12	395	33.42	819	31.05
40–80%	505	34.68	369	31.22	874	33.13
> 80%	525	36.06	418	35.36	943	35.75
Marital status (n %)						
Married/de facto	904	62.09	782	66.16	1686	63.91
Single/divorced/widowed	393	26.99	363	30.71	756	28.66
Interpreter (n %)						
No	1287	88.39	1053	89.09	2340	88.70
Yes	92	6.32	112	9.48	204	7.73
Missing	77	5.29	17	1.44	94	3.56
Tumour stream (n %)						
Head and neck	62	4.26	49	4.15	111	4.21
UGI	83	5.70	134	11.34	217	8.23
CRC	124	8.52	147	12.44	271	10.27
Lung	108	7.42	209	17.68	317	12.02
Other	57	3.91	29	2.45	86	3.26
Skin	49	3.37	49	4.15	98	3.71
Breast	214	14.70	175	14.81	389	14.75
Genitourinary	148	10.16	97	8.21	245	9.29
CNS	60	4.12	36	3.05	96	3.64
Haematological	551	37.84	257	21.74	808	30.63

*ATSI* Aboriginal or Torres Strait Islander status, *SEIFA* Socio-economic indexes for areas, *UGI* upper gastrointestinal, *CRC* colorectal cancer, *CNS* central nervous system

### Characteristics associated with ED presentation

Findings reported below with regard to characteristics associated with ED presentations are presented in accordance with Andersen’s Behavioural Model of Health Service Utilisation.

Being born outside of Australia was the only socio-demographic factor associated with ED presentation (AOR 1.322, 95% CI 1.109, 1.576). Patients diagnosed with lung cancer had more than three times the odds (AOR 3.727; 95% CI 2.781, 4.996) of making an unplanned ED presentation than patients diagnosed with hematological cancers (Table 2).

**Table 2:** Socio-demographic and disease related factors associated with ED presentation within any 28-day given period after SACT

	B	S.E.	Wald	df	*p* value	OR	95% CI		Effect size
							Lower	Upper	
Age	.004	.003	1.306	1	.253	1.004	.997	1.010	trivial
Gender									
Male	−.078	.098	.636	1	.425	.925	.763	1.121	trivial
Female (ref)									
Country of birth									
Other than Australia	.279	.090	9.716	1	.002*	1.322	1.109	1.576	small
Australia (ref)									
Marital status									
Single/divorced/widowed	.092	.093	.988	1	.320	1.097	.914	1.315	trivial
Married/de facto (ref)									
SEIFA (VIC)									
0–40%	.032	.104	.097	1	.756	1.033	.843	1.266	trivial
40–80%	−.082	.103	.642	1	.423	.921	.753	1.127	trivial
> 80% (ref)			1.268	2	.530				
Tumour stream									
Head and neck	.455	.212	4.622	1	.032*	1.577	1.041	2.389	small
UGI	1.074	.168	41.101	1	<.001**	2.927	2.108	4.064	medium
CRC	.767	.148	26.834	1	<.001**	2.154	1.611	2.879	small
Lung	1.316	.149	77.458	1	<.001**	3.727	2.781	4.996	large
Other	.136	.251	.292	1	.589	1.145	.700	1.873	trivial
Skin	.723	.227	10.179	1	.001*	2.060	1.321	3.211	medium
Breast	.365	.144	6.443	1	.011*	1.441	1.087	1.911	small
Genitourinary	.231	.159	2.104	1	.147	1.260	.922	1.721	small
CNS	.253	.234	1.176	1	.278	1.288	.815	2.036	small
Haematological (ref)			110.128	9	<.001**				
Constant	−.893	.224	15.828	1	<.001	.409			

* *p* < .05 ** *p* < .001

### Characteristics associated with frequent ED presentations

Patients diagnosed with genitourinary cancers had twice the odds (AOR 2.035, 95% CI 1.053, 3.934) of making two or more ED presentations within any given 28 day period after receiving cancer treatment. This was the only factor found to be independently related to making multiple ED presentations within any given 28 day period after receiving SACT (Table 3).

**Table 3:** Socio-demographic and disease related factors associated with two or more ED presentations within any 28-day given period after SACT

	B	S.E.	Wald	df	*p* value	OR	95% CI		Effect size
							Lower	Upper	
Age	.002	.007	.092	1	.762	1.002	.989	1.016	trivial
Gender									
Male	−.083	.209	.159	1	.690	.920	.611	1.385	trivial
Female (ref)									
Country of birth									
Other than Australia	.024	.194	.015	1	.901	1.024	.701	1.497	trivial
Australia (ref)									
Marital status									
Single/divorced/widowed	−.278	.211	1.740	1	.187	.757	.500	1.145	small
Married/de facto (ref)									
SEIFA (VIC)									
0–40%	−.191	.231	.687	1	.407	.826	.525	1.298	small
40–80%	.020	.221	.008	1	.930	1.020	.661	1.574	trivial
> 80% (ref)			.992	2	.609				
Tumour stream									
Head and neck	−.309	.565	.299	1	.584	.734	.243	2.220	small
UGI	.072	.343	.045	1	.833	1.075	.549	2.104	trivial
CRC	−.127	.347	.133	1	.716	.881	.446	1.740	small
Lung	.163	.296	.304	1	.582	1.177	.659	2.105	trivial
Other	.284	.578	.242	1	.623	1.329	.428	4.126	small
Skin	.500	.442	1.284	1	.257	1.649	.694	3.919	small
Breast	−.255	.358	.505	1	.477	.775	.384	1.564	small
Genitourinary	.711	.336	4.467	1	.035*	2.035	1.053	3.934	medium
CNS	.666	.472	1.993	1	.158	1.947	.772	4.910	medium
Haematological (ref)			10.229	9	.332				
Constant	−2.049	.502	16.673	1	<.001	.129			

* *p* < .05 ** *p* < .001

Being born outside of Australia was significantly associated with making frequent (four or more within an 12 month period) ED presentations within 28 days of receiving SACT (AOR 1.820, 95% CI 1.178, 2.810, *p* < .01) (Table 4).

**Table 4:** Socio-demographic and disease related factors associated with making frequent (≥ 4 within any 12 month period) ED presentations within 28 days of receiving SACT

	B	S.E.	Wald	df	*p* value	OR	95% CI		Effect size
							Lower	Upper	
Age	.005	.008	.403	1	.525	1.005	.989	1.021	trivial
Gender									
Male	−.096	.230	.176	1	.675	.908	.579	1.425	trivial
Female (ref)									
Country of birth									
Other than Australia	.599	.222	7.293	1	<.01*	1.820	1.173	1.942	small
Australia (ref)									
Marital status									
Single/divorced/widowed	.074	.227	.105	1	.764	1.076	.690	1.680	trivial
Married/de facto (ref)									
SEIFA (VIC)									
0–40%	−.111	.259	.184	1	.668	.895	.539	1.486	trivial
40–80%	.085	.257	.109	1	.741	1.088	.658	1.800	trivial
> 80% (ref)			.605	2					
Tumour stream									
Head and neck	−1.585	1.038	2.329	1	.127	.205	.027	1.569	large
UGI	.462	.340	1.852	1	.174	1.587	.816	3.088	small
CRC	.498	.326	2.335	1	.127	1.645	.869	3.115	small
Lung	.038	.326	.014	1	.907	1.039	.549	1.968	trivial
Other	.170	.654	.068	1	.795	1.185	.329	4.269	trivial
Skin	−1.451	1.038	1.955	1	.162	.234	.031	1.792	large
Breast	−.548	.432	1.612	1	.204	.578	.248	1.347	small
Genitourinary	.176	.399	.195	1	.659	1.193	.545	2.609	trivial
CNS	−1.196	1.043	1.314	1	.252	.302	.039	2.337	large
Haematological (ref)			14.981	9	.091				
Constant	−2.886	.592	23.766	1	.000	.065			

* *p* < .05 ** *p* < .001

### Characteristics associated with place of discharge

Increasing age was significantly associated with being admitted to the inpatient area (AOR 1.014, 95% CI 1.004, 1.024, *p* < .01) for further care. Patients diagnosed with breast cancer (AOR 2.176, 95% CI 1.387, 3.416, *p* < .01) had more than twice the odds of being discharged home from the ED than being admitted to an inpatient area (Table 5).

**Table 5:** Socio-demographic and disease related factors associated with discharge from ED to primary place of residence

	B	S.E.	Wald	df	*p* value	OR	95% CI		Effect size
							Lower	Upper	
Age	.014	.005	7.754	1	.005*	1.014	1.004	1.024	trivial
Gender									
Male	−101	.160	.401	1	.527	1.107	.809	1.236	small
Female (ref)									
Country of birth									
Other than Australia	.096	.145	.435	1	.510	1.100	.828	1.207	small
Australia (ref)									
Marital status									
Single/divorced/widowed	−.058	.150	.151	1	.698	.944	.704	1.421	small
Married/de facto (ref)									
SEIFA (VIC)									
0–40%	.303	.167	3.304	1	.069	1.354	.977	1.877	small
40–80%	.254	.167	2.307	1	.129	1.209	.929	1.790	trivial
> 80% (ref)			3.911	2	.141				
Tumour stream									
Head and neck	−.222	.347	.409	1	.522	.801	.406	1.582	small
UGI	.373	.274	1.853	1	.173	1.453	.402	2.487	small
CRC	−.399	.230	3.014	1	.083	.671	.428	1.053	small
Lung	.161	.228	.501	1	.479	1.175	.752	1.836	small
Other	.653	.565	1.338	1	.247	1.922	.635	5.815	medium
Skin	.377	.400	.887	1	.346	1.457	.666	3.191	small
Breast	−.778	.230	11.431	1	.001*	.459	.293	.721	medium
Genitourinary	.155	.298	.269	1	.604	1.167	.651	2.092	trivial
CNS	−.243	.398	.372	1	.542	.784	.359	1.712	small
Haematological (ref)			27.564	9	.001*				
Constant	.108	.360	.090	1	.765	1.114			

* *p* < .05 ** *p* < .001

## Discussion

This study has identified a number of factors associated with unplanned ED presentation after receiving SACT in a DOU. Predisposing factors such as country of birth and age, and disease related factors such as diagnosis were found to be independently associated with ED presentation.

Nearly 45% of all patients in this study who were treated for cancer in the DOU presented to the ED within 28 days. The number of patients who presented to the ED after receiving SACT in this study is higher than that reported in other Australian studies, which have ranged between 30 and 45% [5, 9, 32], while international studies have reported ED utilization rates as high as 83% in patients receiving SACT [10]. Other studies exploring ED utilization rates have however used a variety of methods to identify patients who have been treated in the DOU and who have subsequently attended the ED, which may explain the variance reported. The setting of this study was a large tertiary referral centre that treats patients with complex and rare cancers as well as patients recruited to Phase 1–4 cancer clinical trials. As such, the case mix of patients may represent more complex or unwell patients who are consequently more likely to present to the ED. Higher rates of ED utilization reported internationally may be explained by the inclusion of patients who have received SACT up to 12 months prior to their indexed ED presentation.

The vast majority of studies that have explored ED presentation in the cancer patient population have focused on factors that may be associated with inpatient admission following an ED presentation. This study is unique in its approach to identifying patients who may be at risk of making an ED presentation based on socio-demographic and disease related characteristics identifiable at the initial treatment phase.

To the best of our knowledge this is the first time Andersen’s Model has been operationalized to explore factors associated with ED presentation in cancer patients receiving SACT in an ambulatory setting. Our work indicates the relevance of Andersen’s Model to explore factors influencing presentation to ED within 28 days of receiving ambulatory-based anti-cancer therapy, and suggests further work is warranted, to explore the relevance and applicability of the model.

### Predisposing factors

Predisposing factors found to be associated with unplanned ED presentation in this study included being born outside of Australia, with over 50% (*n* = 548/1075) of patients in this study born outside of Australia presenting to the ED within 28 days of receiving SACT. A similar Australian study by Craike et al., reported 37.7% of patients treated in the DOU who were born in a non-English speaking country presented to the ED on at least one occasion [33]. In the present study, of the 1075 (40.8%) patients born outside of Australia, 300 (27.9%) recorded a primary language other than English, and of those 199 (66.3%) needed an interpreter for in-hospital communication. In a recent U.S. study by Ngai et al.*,* a significant relationship was demonstrated between the general population of ED attenders with limited English proficiency, and making unplanned ED re-presentations within 72 h [34]. Our study found no association between English proficiency and frequent ED presentations, in both univariate and multivariate analysis. However, we found that limited English proficiency was associated with making at least one ED presentation after receiving SACT (*p* < .001), but not with making frequent ED presentations (data not shown). The significant association between being born outside of Australia and making an unplanned ED presentation in this study suggests that increased risk of ED presentation may not be based on language or communication barriers alone, but may be due to additional factors such as being unfamiliar with the Australian health care system. Stage of disease may also be an important factor.

### Enabling factors

A number of international studies have identified several factors that may be associated with ED presentation such as lower socio-economic status [13, 33, 35]. In our study no factors likely to increase likelihood of or mitigate against ED presentation were identified.

### Need factors

Disease related factors found to be associated with an ED presentation (head and neck, UGI, CRC, lung, skin or breast cancer diagnosis) may represent true symptom severity and need for health care but may also indicate levels of unmet supportive care need. In 2013, Aprile et al., reported that 21.6% of unplanned hospital presentations made by cancer patients were due to the patients’ desire for reassurance from their treating specialist [12]. Public use and perception of EDs were explored in an Australian study by Fitzgerald et al.*,* which reported that the main reasons for ED use by the general population were perceived severity of illness, unavailability of alternative health services, and the perception that EDs were able to provide a higher standard of care [36]. More recently, Phillip et al.*,* reported that patients with advanced cancer frequently presented to the ED as a means of accessing specialist oncology care, to receive treatment for worsening symptoms, or symptoms that they had been instructed to attend the ED for, such as a fever [4]. To the best of our knowledge, no studies have explored the decision-making process behind ED presentations for patients receiving active cancer treatment.

Results from this study have shown that patients who were of an older age were significantly more likely to be admitted to either the SSU or to an inpatient unit, than be discharged home following an ED presentation. This may suggest the presence of other comorbidities alongside a diagnosis of cancer, and thus a higher level of care complexity or clinical acuity [3, 37, 38]. This finding is consistent with international literature that has previously identified a significant relationship between older age and inpatient admission following an ED presentation [39, 40].

Patients diagnosed with breast cancer were more likely to be discharged home than be admitted to an inpatient area after an ED presentation. Previous studies have explored risk factors for inpatient admission following an ED presentation, but no studies were identified that explored the factors associated with being discharged home from the ED. Patients with early stage breast cancer are commonly treated with adjuvant chemotherapy and subsequently experience considerable treatment related toxicities and are known to make higher numbers of unplanned hospital visits [41, 42]. This may explain the higher frequency of ED presentations seen in this study, but also why ED presentation by such patients is unlikely to result in admission.

Patients with haematological cancers are known to have high rates of ED utilization due to the acuity and complexity of these diseases and their treatments [8, 37]. In this study, patients diagnosed with hematological cancers made the largest number of ED presentations, but which are comparatively low to the number of patients treated for hematological cancers in the DOU. At the study site patients with haematological cancers have timely access to specialist haematology nurse coordinators who provide telephone support during business hours. This may explain the low numbers of ED presentations by haematology patients in our study. Equivalent nurse coordinators are only available for patients diagnosed with solid tumours who are receiving oral anti-cancer therapies or concurrent chemotherapy and radiotherapy. Outside of these treatment modalities, patients only recourse is to contact the DOU and speak a senior oncology nurse where they are provided with telephone advice or advised to present to the ED for further assessment.

## Conclusion

Findings from this retrospective analysis suggest that it may be possible to proactively identify patients at greatest risk of making an unplanned ED presentation after receiving SACT in a DOU setting.

Further research is needed to test the clinical utility of socio-demographic and disease related factors, health behaviors and outcomes of health service use that are not currently routinely collected as a component of administrative health datasets, but which may be predictive of an unplanned ED presentation after receiving SACT.

In this study, disease related factors such as being diagnosed with head and neck, upper gastrointestinal, colorectal, lung, melanoma and breast cancers were significantly related to risk of ED presentation after receiving SACT in the DOU setting, and were a stronger predictor of ED presentation than socio-demographic characteristics. These findings could be used to proactively identify patients for targeted follow up.

Drawing on these data and best available evidence, findings from this study are being used to develop, implement and evaluate a nurse-led Symptom and Urgent Review Clinic service that will provide proactive follow-up and support to patients identified as high risk of making unplanned ED presentations. The clinic will include standardized telephone triage and physical assessment for cancer patients experiencing disease or treatment-related symptoms.

### Limitations

The limitations of the administrative databases make drawing conclusions about why certain cohorts are more likely to make unplanned ED presentations challenging. Important variables that may be associated with ED presentation such as stage of disease and those receiving multi-modality treatment are not currently captured in these datasets in Victoria, Australia.

This study was conducted at a single site and therefore may not generalizable to other healthcare settings. The models of care to support patients outside the hospital setting who experience cancer and treatment related toxicities, the case mix of patients treated at individual settings and the difference in health systems internationally means that risk factors for ED presentation may differ according to local structures.

## Data Availability

The datasets used and/or analysed during the current study are available from the corresponding author on reasonable request.

## Abbreviations

DOU: Day Oncology Unit
ED: Emergency Department
SACT: Systemic anti-cancer therapy
